# Primary posterior mediastinal angiolipoma: a case report

**DOI:** 10.1093/jscr/rjab168

**Published:** 2021-05-06

**Authors:** Daisuke Nakamura, Nobutaka Kobayashi, Masahisa Miyazawa, Hidetoshi Satomi, Nobumaro Ihara, Mayu Tsunoda

**Affiliations:** Department of Thoracic Surgery, Japanese Red Cross Society Nagano Hospital, 5-22-1 Wakasato, Nagano, Japan; Department of Thoracic Surgery, Japanese Red Cross Society Nagano Hospital, 5-22-1 Wakasato, Nagano, Japan; Department of Thoracic Surgery, Japanese Red Cross Society Nagano Hospital, 5-22-1 Wakasato, Nagano, Japan; Department of Pathology, Japanese Red Cross Society Nagano Hospital, 5-22-1 Wakasato, Nagano, Japan; Department of Radiology, Japanese Red Cross Society Nagano Hospital, 5-22-1 Wakasato, Nagano, Japan; Department of Radiology, Japanese Red Cross Society Nagano Hospital, 5-22-1 Wakasato, Nagano, Japan

**Keywords:** angiolipoma, embolization, mediastinum, video-assisted thoracic surgery

## Abstract

An angiolipoma is a benign tumor, and a primary mediastinal angiolipoma is extremely rare. Herein, we describe the presentation and management of a posterior mediastinal angiolipoma in a woman with loss of consciousness. Chest computed tomography (CT) revealed a contrast-enhancing mass in the right posterior mediastinum, with intercostal arterial blood supply identified on three-dimensional reconstruction CT (3D-CT). Magnetic resonance imaging revealed a fatty component. Pre-operative embolization of the supplying intercostal artery was performed to reduce intraoperative bleeding. Mass resection was performed using video-assisted thoracic surgery. Histopathology confirmed angiolipoma diagnosis. Although rare, a posterior mediastinum angiolipoma should be considered a possibility; 3D-CT and pre-operative embolization may be useful in the surgical treatment of hypervascular mediastinal tumors, such as angiolipomas.

## INTRODUCTION

Angiolipomas are rare, benign, soft tissue tumors comprising mature fatty tissue and blood vessels, occurring in the subcutaneous tissue and extremity muscles [1]. Primary mediastinal angiolipomas are extremely rare [2–5]. We present an extremely rare posterior mediastinal angiolipoma, which we successfully resected using VATS post-embolization.

## CASE REPORT

**Figure 1:**
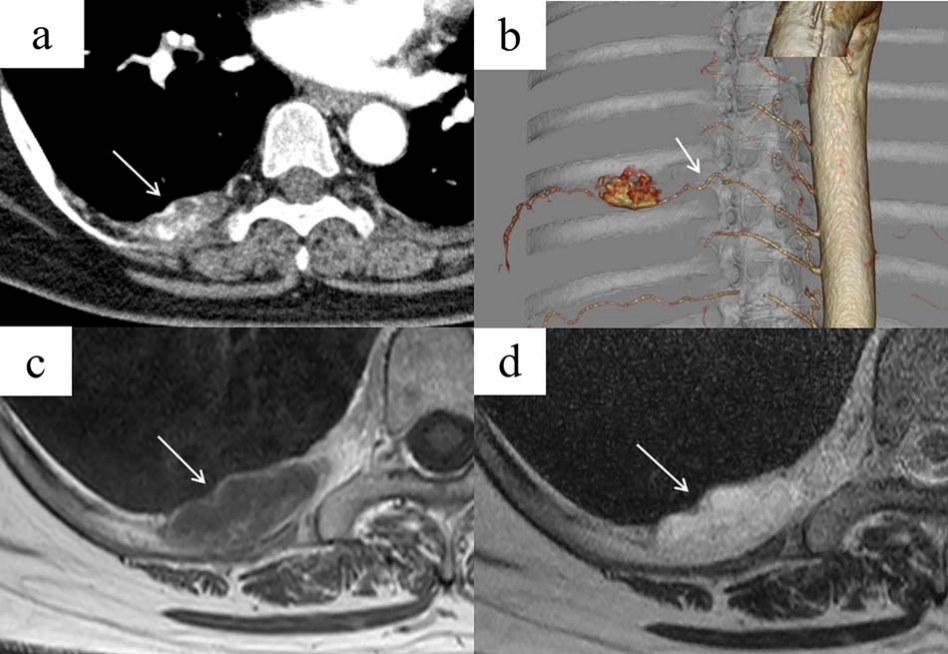
(**a**) Contrast-enhanced chest CT showing a right posterior mediastinal mass measuring 35 mm in diameter with heterogeneous contrast at the T7–T8 level, without extension into the spinal canal (arrow). (**b**) Three-dimensional reconstruction CT showing a feeding artery to the mass arising from the intercostal artery (arrow). (c, d) MRI showing low signal intensity on T1-weighted images but a bright signal on T2-weighted images (arrows). A high-signal area on T1-weighted MRI is suggestive of a fatty component inside the tumor. (**c**) T1-weighted image. (**d**) T2-weighted image.

**Figure 2:**
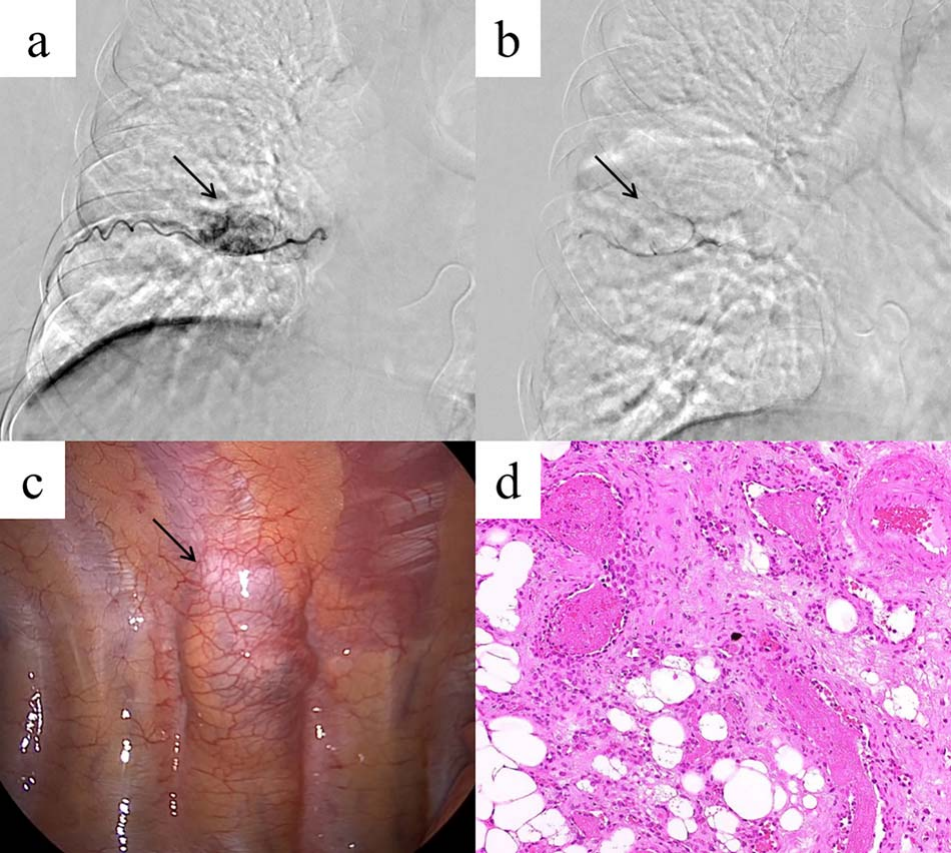
(**a**) Angiography showing a feeding vessel flowing into the mass from the right eighth intercostal artery and tumor staining (arrow). (**b**) Angiography after embolization showing disappearance of the tumor staining (arrow). (**c**) Intra-operatively, the mass is a soft tumor with a fatty component, but there is no invasion of the surrounding tissue (arrow). (**d**) Microscopic examination of the resected tumor reveals mature adipocytes and multiple small vessels (hematoxylin and eosin stain, original magnification ×200).

A 48-year-old woman was brought to our hospital owing to loss of consciousness; she had no major disease history, an unremarkable physical examination and normal laboratory findings. Contrast-enhanced chest CT revealed a mass 35 mm in diameter, with heterogeneous contrast, in the right posterior mediastinum at the thoracic T7–T8 level (Fig. 1a). The mass was not considered to have extended into the surrounding tissues or the spinal canal. An intercostal arterial feeder to the mass was observed on 3D-CT (Fig. 1b). Magnetic resonance imaging (MRI) revealed low T1-weighted and high T2-weighted signals in the mediastinal mass. A high-signal area on T1-weighted MRI suggested a fatty component inside the mass (Fig. 1c and d). We suspected a schwannoma or hemangioma or lipoma preoperatively and planned surgical resection Based on evidence of hypervascularity of the mass, preoperative embolization was planned to prevent unexpected massive intraoperative bleeding. On angiography, a feeding vessel from the right eighth intercostal artery to the mass was identified, with associated staining of the mass (Fig. 2a). We selectively embolized this feeding vessel using gelatin, resulting in the associated disappearance of staining (Fig. 2b). On Day 1 post-embolization, the tumor was resected using VATS. Intraoperatively, a soft mass with a fatty component and no surrounding tissue invasion was confirmed (Fig. 2c). The tumor was completely resected, in 96 min with minimal intraoperative blood loss. Microscopy revealed mature adipocytes, multiple small vessels and no malignancies (Fig. 2d). Histopathology confirmed a posterior mediastinal angiolipoma. The patient’s post-operative course was uneventful, with no recurrence at the 6-month follow-up post-surgery.

The patient provided informed consent for the publication of this report.

## DISCUSSION

An angiolipoma is a rare, benign, mesenchymal tumor, accounting for 5–17% of all lipomas [1], comprising mature fatty tissues and blood vessels, and developing in the subcutaneous tissue and upper extremity muscles in young adults [1]. A mediastinal angiolipoma is extremely rare [2–5]. Previous reports of posterior mediastinal angiolipoma have shown the possibility of tumor extension to the intervertebral foramen or spinal canal [2, 4, 5] with neurological symptoms [4]. Owing to the fatty component observed on imaging and the location of the mass in the posterior mediastinum, liposarcoma, lipoma and neurogenic tumor were considered during the pre-operative differential diagnosis. However, Gorospe *et al.* reported a radiologically lipid-poor posterior mediastinal angiolipoma [2]; thus, consideration of this diagnosis is warranted. In that case, similar to ours, the mediastinal angiolipoma was surgically resected, with a good post-operative course.

No evidence of tumor invasion into the surrounding tissues indicated a benign tumor. The hypervascularity and location of the mass preoperatively suggested a hemangioma or neurogenic tumor. Retrospectively, the preoperative MRI suggested a lipoma, liposarcoma or angiolipoma owing to mixed fat components. Although extremely rare, the differential diagnosis of mediastinal tumors should include angiolipoma.

Surgical resection was necessary; however, massive bleeding during surgery was expected because the tumor was hypervascular, and a feeding vessel arising from the intercostal artery was detected on 3D-CT. Fujiu *et al.* reported a case of angiolipoma with massive intra-operative bleeding in excess of 3000 mL [6]. Massive bleeding during biopsy for an angiomyolipoma, similar to an angiolipoma, was previously reported [7]. Pre-operative embolization was shown to be useful for posterior mediastinal angiolipoma [2]. To the best of our knowledge, ours is the first report of a feeding vessel, supplying the mediastinal angiolipoma, on 3D-CT, which allowed us to perform preoperative embolization and safely complete the surgery with VATS.

In conclusion, this is the second reported posterior mediastinal angiolipoma treated with pre-operative embolization followed by resection. Although rare, an angiolipoma should be considered in the differential diagnosis of posterior mediastinal tumors. Moreover, 3D-CT might be useful to identify feeding vessels to the posterior mediastinal tumor, thus assisting in treatment planning. Preoperative embolization for hypervascular tumors is useful in reducing intra-operative blood loss, with subsequent successful resection using VATS.
